# What is depression? Psychiatrists’ and GPs’ experiences of diagnosis and the diagnostic process

**DOI:** 10.3402/qhw.v9.24866

**Published:** 2014-11-06

**Authors:** Annette S. Davidsen, Christina F. Fosgerau

**Affiliations:** 1 The Research Unit for General Practice and Section of General Practice, Department of Public Health, University of Copenhagen, Copenhagen, Denmark; 2 Department of Scandinavian Studies and Linguistics, University of Copenhagen, Copenhagen, Denmark

**Keywords:** Depression, clinical utility, rating scales, collaborative care, psychiatry, general practice

## Abstract

The diagnosis of depression is defined by psychiatrists, and guidelines for treatment of patients with depression are created in psychiatry. However, most patients with depression are treated exclusively in general practice. Psychiatrists point out that general practitioners’ (GPs’) treatment of depression is insufficient and a collaborative care (CC) model between general practice and psychiatry has been proposed to overcome this. However, for successful implementation, a CC model demands shared agreement about the concept of depression and the diagnostic process in the two sectors. We aimed to explore how depression is understood by GPs and clinical psychiatrists. We carried out qualitative in-depth interviews with 11 psychiatrists and 12 GPs. Analysis was made by Interpretative Phenomenological Analysis. We found that the two groups of physicians differed considerably in their views on the usefulness of the concept of depression and in their language and narrative styles when telling stories about depressed patients. The differences were captured in three polarities which expressed the range of experiences in the two groups. Psychiatrists considered the diagnosis of depression as a pragmatic and agreed construct and they did not question its validity. GPs thought depression was a “gray area” and questioned the clinical utility in general practice. Nevertheless, GPs felt a demand from psychiatry to make their diagnosis based on instruments created in psychiatry, whereas psychiatrists based their diagnosis on clinical impression but used instruments to assess severity. GPs were wholly skeptical about instruments which they felt could be misleading. The different understandings could possibly lead to a clash of interests in any proposed CC model. The findings provide fertile ground for organizational research into the actual implementation of cooperation between sectors to explore how differences are dealt with.

Depression is an increasing challenge worldwide (Marcus, Yasama, Van Ommeren, & Chisholm, 2012) with a lifetime prevalence of about 20% (Kessler et al., 2005). The diagnosis of depression is defined in ICD-10 and DSM-V through a complex process of professional dispute among psychiatrists. Most patients with depression are, however, treated exclusively in general practice (Goldberg & Lecrubier, 1995). Psychiatric researchers often argue that general practitioners (GPs) do not recognize sufficient number of patients with depression, that they treat too few patients, and that treatment is insufficient (Cepoiu et al., 2008; Davidson & Meltzer-Brody, 1999; Kocsis et al., 2008; Lecrubier, 2007; Mitchell, Vaze, & Rao, 2009). In different countries, governments and administrators struggle to deal with the challenges of how to improve depression care (Gunn et al., 2010; Telford, Hutchinson, Jones, Rix, & Howe, 2002).

Collaborative care (CC) models are mentioned in different guidelines as an effective solution to the problem (Kennedy, Lam, Parikh, Patten, & Ravindran, 2009; National Institute for Clinical Excellence, 2009). Such models have shown to be effective in the USA (Archer et al., 2012) but have shown lesser effect in the UK (Richards et al., 2013). In Denmark, a project has just been launched to study such a CC model (Collabri) (Eplov et al., 2014). In this model, psychiatric nurses, employed in psychiatry, and supervised by psychiatrists will treat patients in general practice.

There is a growing awareness that effective implementation of such collaborative treatment models is dependent upon a shared understanding among the professionals (May, 2013). Successful implementation of a CC model demands a shared agreement about what is meant by the term depression and how the diagnostic process should be dealt with (Gunn et al., 2010).

A shared understanding does not necessarily exist between GPs and psychiatrists. GPs have been shown often to have different views of depression than psychiatrists (Chew-Graham, Mullin, May, Hedley, & Cole, 2002; Dowrick, 2009a, 2009b; Gask, Klinkman, Fortes, & Dowrick, 2008). Primary care authors are concerned with the uncertainty of the diagnosis of depression (Dowrick, 2009a, 2009b). Different studies show that, in general practice, patient context or background variables become important in establishing mental health problems (Gask et al., 2008; Schumann, Schneider, Kantert, Löwe, & Linde, 2012; Van Rijswijk, Van Hout, Van de Lisdonk, Zitman, & Van Weel, 2009) and that depression is often regarded as part of normal life events (Chew-Graham et al., 2002). The GP is involved in the patient’s overall situation and the symptoms are interpreted on the background of the patient’s medical and social context (Armstrong & Earnshaw, 2004). GPs may, therefore, view depression as an everyday problem of practice, rather than as an objective diagnostic category (Chew-Graham, May, & Headley, 2000; Chew-Graham et al., 2002).

General practice researchers stress that the diagnoses, guidelines, and rating scales created in psychiatry are not validated in primary care and not directly transferrable to general practice (Dowrick et al., 2009; Kendrick, 2000; Thorsen, la Cour, & Brodersen, 2003). It is still unclear whether there are qualitative differences between depression seen in general practice and in psychiatry. It has been shown that the mood symptoms of depression are more likely to be presented by patients in psychiatry, whereas fatigue is more likely to be presented in general practice (Suh & Gallo, 1997). In addition, studies which have not found any qualitative differences have focused on patients already diagnosed with depression (Gaynes et al., 2007; Thombs et al., 2011).

Most patients with depression present in primary care with somatic symptoms. Moreover, somatic symptoms are strongly associated with coexisting depression, and some of these patients have medically unexplained symptoms (Kroenke, 2003) Furthermore, primary care patients with depression are more likely to suffer from disabling medical conditions., and duration of depression is longer in primary care patients and more often has a chronic course (Vuorilehto, Melartin, Rytsala, & Isometsa, 2007).

The debate about the diagnosis of depression has, however, not only taken place in primary care. For decades there has also been a theoretical debate within psychiatry about the diagnosis of depression (Berrios, 1988, 1993, 1999; Frances & Egger, 1999; Horwitz & Wakefield, 2007). Pilgrim and Bentall maintained that no working definition of depression is offered at all and that the condition is based on a list of symptoms giving the concept a self-evident validity. They argued that depression comprises the common cold of psychopathology and that the medical diagnostic approach to depression individualizes underlying social processes possibly leading to a professional reification of human misery (Pilgrim & Bentall, 1999).

Some psychiatric authors maintain that the psychiatric view of depression does not necessarily correspond with patients’ understanding (Weich, Morgan, King, & Nazareth, 2007), leading to a gap between psychiatric concepts of depression and the views of patients (Dura-Vila, Littlewood, & Leavey, 2011; Erdal, Singh, & Tardif, 2011; Karasz, 2008). Some consider that in contemporary psychiatry the patient’s clinical presentation is viewed, not as a form of communication, but as a manifestation of disease with the implication that there is no equivalence between psychiatric categories generated through a complex process of professional dispute and patients’ understanding (Karasz, 2008). GPs’ views have been shown to be more similar to patients’ than to psychiatrists’ views (Rogers, May, & Oliver, 2001).

Some psychiatrists maintain that we do not have a clear idea of how to fix the threshold for mental disorders such as depression. Nor do we have a firm grasp on how to differentiate between these disorders and reactions to adverse life events (Maj, 2007). Maj considered some of the reasons for this lack of clarity and understanding to be the definition of mental disorders by current operational diagnostic criteria wrongly encompassing natural reactions to life events (Maj, 2008). With the latest version of the DSM classification (DSM-V) some think that this could lead to overdiagnosis and “medicalizing of unhappiness” (Dowrick & Frances, 2013).

We realized that the theoretical debate about the diagnosis of depression has taken place both in general practice and in psychiatry, and that there has also been a joint initiative to discuss the subject (Dowrick & Frances, 2013). However, we did not know how clinical psychiatrists understand the concept and whether their views differ from those of the GPs. If there is a difference, it might constitute a challenge to the implementation of a CC model. We therefore aimed to explore GPs’ and clinical psychiatrists’ understandings of depression; how psychiatrists and GPs conceptualized depression and experienced their approach to the diagnostic process of the condition in their clinical work.

## Method

We chose a qualitative approach with in-depth interviews with purposively selected psychiatrists and GPs in Denmark (Gubrium & Holstein, 2002; Patton, 2002).

### Data collection

Participants were selected purposively with stepwise recruiting until theoretical saturation was achieved (Strauss & Corbin, 1998). The aim was to cover the range of variation among the two types of professionals. It was presumed that this was achieved through sampling psychiatrists from different institutional backgrounds and different regions, and GPs from different types of practice, whether they were in urban or rural settings, and in partnerships or single-handed general practice. Moreover, participants were sampled with diversity regarding age and sex. Information about GPs and psychiatrists in specialist practice was obtained from a Danish website: www.sundhed.dk, and information about hospital psychiatrists from the leading consultants of the departments.

Eleven psychiatrists and 12 GPs were sampled from two different regions in Denmark. Interviews were conducted from June 2010 until July 2012 by the first author, herself a physician with experience from both general practice and psychiatry. The intention was to address participants’ understanding of depression, and their experience of the diagnostic process in their clinical work (Smith & Osborn, 2003). All participants worked in the public Danish health care system. The diagnostic criteria for depression in Denmark, and other European countries, are the criteria described in ICD-10. We wanted to explore the understanding of these depression criteria in the two groups of physicians who treat patients with depression, how they experienced that depression showed itself in their patients, and how they decided if they would call it a depression.

We had designed an interview guide beforehand pursuing open-ended questions that mirrored the above mentioned purpose and topics of the study. The opening question was what the physicians thought constituted the condition of depression. Further questions dealt with the physicians’ experience of how depression showed itself in their patients and with their experience of the process of diagnosing depression. In addition, the interviewer encouraged narrative accounts about specific patients, which were intended to elicit more contextualized information and perceptions from the participants.

Both groups of participants were generally willing to participate. As regards hospital psychiatrists, some declined due to recent restructuring of their departments to achieve greater specialization, involving job rotation and much administrative work. A few GPs and psychiatrists in specialist practice declined due to lack of time. We did, however, end up with a maximum variation sample. The interviewer had no personal or professional relationship with the participants. There was an equal gender distribution between both groups. Age range was comparable: for psychiatrists, 45–62 years and for GPs, 43–66 years. Six psychiatrists worked in public hospital-based outpatient clinics, three in university hospitals, three in smaller hospitals, and five in specialist practice with collective agreement with the Danish health service (no payment from patients). The interviews lasted 35–56 min and took place at each physician’s workplace. We recorded all interviews digitally and transcribed them verbatim.

### Data analysis

We used Interpretative Phenomenological Analysis (IPA) for detailed structural analysis of the interviews (Smith, Flowers, & Larkin, 2009). IPA runs through a two-stage interpretation process, and could be called double hermeneutic: participants make sense of their world, and the researcher makes sense of the participants’ making sense (Smith & Osborn, 2003). In this way IPA combines an empathic hermeneutics with a questioning hermeneutics (Langdridge, 2007b).

During the initial analysis we read each interview transcript repeatedly to get an overall impression. During the second stage we highlighted text parts for every participant with words or phrases that reflected the content of the participant’s account. The further iterative analytic process led to increasingly conceptual terms, which finally led to themes for each individual interview. We carried out the same analytic process with each transcript and compiled a list of themes with illustrative extracts from each participant. During the final step, we identified higher order “super-ordinate” themes that represented overriding themes from all the interviews.

The two groups of participants differed markedly in their use of language, in the particular phenomena they described as important, and in their description of their approach. Therefore their experiences could not easily be represented through common themes. Nevertheless, their experiences related to the same phenomena. The experiences were interrelated and could be placed along three polarities, which we used instead of stand-alone themes. In addition, we found that participants differed in their narrative styles and so we included elements of narrative analysis (Riessman, 2008). Both methods focus on storied accounts, human agency, imagination, and how an incident is storied. Therefore the combination of phenomenological and narrative methods can be fruitful (Davidsen, 2013; Langdridge, 2007a).

The interviews were read by a group of researchers, one being a physician and the other three language psychologists. The analysis was first carried out by the first author and thereafter discussed in the group of researchers to ensure a common understanding of the findings.

### Ethics

Participants received both written and oral information about the study and gave verbal informed consent. They were informed of their right to withdraw from the study at any time and they were guaranteed confidentiality of the information given in the interviews. During the interview and subsequent interpretation ethical principles for qualitative studies were taken into account (Fog, 2004; Kvale, 2003). In the article anonymity is ensured in the illustrating examples.

## Results

### Different stories

The two groups of participants differed completely in their use of language when talking about depression and patients with depression. Psychiatrists used medical language and focused almost solely upon symptoms of depression and the agreed diagnostic criteria for the diagnosis. They talked in general terms about groups of patients whereas GPs’ focus was specifically on individual depressed patients. Psychiatrists sometimes used a depersonalizing language and designated patients with depression as “depressions.” Additionally, the two groups differed markedly in the types of stories they told about patients. Psychiatrists mainly told a medical story with diagnostic problems and often focused on stories where they found that the patient had been previously incorrectly diagnosed. These stories were very short.

The GPs’ stories were longer than those of psychiatrists and with little use of medical language. GPs’ stories were about individual patients with losses and traumas, and the stress caused by social conditions. The GPs emphasized that knowledge of the patients’ background was important for the assessment of the situation. When GPs told patient stories, these were often complex and with many details. The stories were about patients who were difficult to put into boxes.

### Polarities

In addition to different narrative styles the two groups’ views on the usefulness and applicability of the diagnosis of depression and of diagnostic instruments, such as rating scales could be categorized as polarities in three different areas: Depression as a “Gray area or a Pragmatic construct”; approaching possible depression in patients by “Exploring the terrain or through a Direct approach”; and “Sure instinct or Instruments to make the diagnosis.”

### Gray area or pragmatic construct

Psychiatrists considered the diagnosis of depression as a pragmatic construct which there is agreement about in the diagnostic criteria. They said that they did not perceive any problems with the diagnosis, which they considered expressed current professional agreement and clinical delimitation of apparently comparable conditions. According to them, diagnosing depression was learned through experience and consensus in the professional environment. They did not question the usefulness of the diagnosis and had no difficulties making it. One psychiatrist expressed it as follows:

Interviewer: Are you ever in doubt about the diagnosis?

Psychiatrist: Very seldom. I have been in psychiatry for many years. Well, I perceive I have the diagnosis in place after so long a time.

GPs often felt in opposition to psychiatry. They referred to “the psychiatric way of viewing depression,” which they could not easily relate to the conditions they saw in general practice. They had many reflections on the diagnostic concept and questioned its clinical utility. Some felt that the “depression border” had moved too far into normality. They described patients’ problems as life-phase problems, existential problems, or problems about identity which they were reluctant to label as depression, even if the patients met the diagnostic criteria.

I talk to really many patients with something which I would call crises or existential problems. But I have difficulties accepting the medical concept of depression because I think—perhaps the sensitivity is OK, but the specificity I think is absolutely hopeless—if I am trying to distinguish from normal life crises.

One quoted Yalom, saying “forget about diagnosis, except for insurance companies.” GPs often considered the condition of depression a “gray area” which was difficult to label. Patients could concomitantly have symptoms from anxiety, depression, somatization, and eating disorders and GPs said they regarded the symptoms as different dimensions of one condition.

There are really many patients, who have eating disorders for example, and anxiety, and depression. And I always say to them, well, you do not have three diseases. You have a condition in you manifesting itself by these different things.

Often GPs found it difficult to distinguish depression from reactions to life circumstances; and because treatment was the same in these cases they considered it more important to find some working points with their patients, independent of diagnosis.

Psychiatrists, on the other hand, focused on the depression and the symptoms of depression as their main target. Problems which the patients might have in their life situation were thereafter also categorized in the diagnostic system, as an added secondary diagnosis.

Psychiatrist: Well, you have to make a symptom diagnosis, and it is a symptom you treat, and you have a primary diagnosis based on that, and if the symptom is depressive then you can call it anything, but you need to have a depression diagnosis somewhere.

Interviewer: that does not tell anything about what causes it or about other characteristics of the depression.

Psychiatrist: there you have your secondary diagnosis, right.

### Exploring the terrain or a direct approach

GPs described how patients came to them with blurred symptoms, tiredness, sadness, and hopelessness, often mixed with bodily symptoms and linked to a story from the patient’s life-world. There was always a phase of clarification where the GP tried to come to an understanding of the patient’s story and symptoms.

So I try to create as safe an atmosphere as possible, so everything can be said. I try to avoid zooming in too quickly—to take time to explore the terrain, before I decide, first of all, on the diagnosis or condition, second, what we should do about it.

Some patients came to their GP because of a breakdown at work and the GPs said that it was difficult to know whether the problem was work-related or if a depression had caused the problem at work. Other patients presented with somatic symptoms and the GPs said that it could take a long time and several visits to get at an understanding of the problem. The true problem, and the weight of it, might be different from what was anticipated when the patient first presented.

Well, I use time as a factor—I mean, I spend time talking to people several times. … and then I’m sometimes just puzzled whether something was serious, and then it wasn’t anyway, and other times it actually goes a lot deeper than I first thought.

GPs said that often the problems were not obvious: “They do not necessarily come in with a label on their forehead saying that it is about this or that.” In addition, GPs perceived that it could be really difficult to convince patients that they were depressed. Some GPs felt that patients considered it an insult if the GP said that the condition was caused by psychological mechanisms. These GPs described the negotiation with patients about the diagnosis as “a mega-great work” on the patients’ motivation, because patients often insisted that somatic disorders or life events were responsible for their problems. In addition, in the GPs’ experience, mental disorder was associated with self-reproach in the minds of some patients.

It can be very difficult to convince them that they—that it is something mental they suffer from. And I also think that it is my duty to tell them, that there is nothing somatic the matter with them, but that something is the matter, and not just turn them down by … it is just something mental, isn’t it, so you brought it on yourself. There is a lot of self-reproach in that.

Psychiatrists described a direct approach to the diagnostic process with questioning the patient about depression symptoms: “Well, it is very concrete, depression and the symptoms you can line up.” Or:

We have a routine procedure, you could say. They get an appointment for a prior conversation, most of them get a rather structured interview, often at least a great part of a PSE,^1^ and then nowadays all of them are assessed by a Hamilton depression scale.

Psychiatrists did not spontaneously offer external factors as influencing or precipitating the condition. They mentioned social factors but these were not something they took action on, and they referred to environmental factors in medical, diagnostic terms, as in the above example, or in general terms not linked to individual patients. One psychiatrist said: “I explain the environmental mechanisms using some statistics and pictures from logical investigations. They show, for example, that stress, abuse, or adverse events can increase the risk of depression.”

### Sure instinct or instruments

GPs said they felt a demand from psychiatry, from guidelines, and from their collective agreement to use instruments to make the diagnosis. The scales had been incorporated into the collective agreement, and GPs were reimbursed for using them. They felt that “the psychiatric way of viewing depression” had forced itself into general practice leading the treatment astray.

We are almost constrained to use this, and in many ways it is a step forward, because it forces you to use a systematic approach, but—and from my point of view, absolutely—it is also in many ways experienced as a strange distortion.

GPs were skeptical about rating scales. They did not feel that the scales were useful in the normal clinical working day because patients did not fit into boxes. A few GPs used scales but said that they often kept the items at the back of their mind. They did not have a list in front of them because this did not fit into a fluid conversation.

Psychiatrists, however, stressed that they did not use scales for making the diagnosis. Here the clinical interview and the clinical impression were more important. As one of them said: “The clinical interview, that is the queen of the clinic.” The clinical impression could even be so strong that it could overrule the diagnostic criteria. However, the psychiatrists had difficulty explaining what this clinical impression covered, besides that it was developed through experience.

Psychiatrist: You see depressions (depressed patients) that are for example just sleep disturbed. You see depressions that are more demented than permitted, but most of it is cognitive disturbances due to the depression. And it can, you do not find justification for that in the diagnostics, you don’t, but you must be attentive …

Interviewer: but you think that it does nevertheless belong to that diagnosis? What is it then—in some way something must tell you that it belongs to that category without actually fitting into ICD-10.

Psychiatrist: Well, you see you just have a sure instinct.

After the diagnosis had been made psychiatrists used instruments to measure where the patient was placed according to severity. Generally, psychiatrists said they could rely on the rating scales regarding the severity of the depression, whereas GPs expressed a greater experience of discrepancy between their clinical impression of severity and the scales. Sometimes the scales did not correspond with the GPs’ “gut feeling.” They said that often a patient who they considered really depressed did not have a high score, and the opposite could also be the case and that in general practice the patient’s condition was often fluctuating.

Well, it’s not so simple, at least not for them, is it a life crisis we’re talking about here? Because ICD-10 or the other one, MDI, can suggest that there’s something wrong, but in the long term it turns out that there’s nothing wrong anyway. It may just have been a time-lapse image, or, you know, a snapshot, and then it wasn’t that significant anyway.

## Discussion

The two groups of physicians differed in their views on the usefulness of the concept of depression and told different stories in different narrative styles about patients with depression. GPs expressed opposition to the “psychiatric way of viewing depression” which they considered a reductionist approach (Stange & Ferrer, 2009). They felt a demand to make the diagnosis based on instruments and felt this as being in opposition to the complexity of the conditions they saw in general practice (Gask et al., 2008; Mitchell et al., 2009).

However, for psychiatrists, the diagnosis did not depend on instruments but on their clinical impressions and their intuition. Psychiatrists relied fully on their clinical impression to distinguish if the patient fitted into the depression category, and thereafter they used instruments to assess the severity. Sometimes their clinical impression even overruled the diagnostic criteria.

In terms of severity GPs likewise thought that scores on rating scales did not correspond to their clinical impressions and that the scales could actually be misleading. Dowrick states that GPs confer higher value on the benefit of their own clinical judgment when assessing patients with depression, than on evidence derived from external sources such as rating scales (Dowrick, 2009b; Van Weel, Van Weel-Baumgarten, & Van Rijswijk, 2009). The rating scales are not validated in general practice and it is still a question whether depressions seen in general practice differ qualitatively from depressions seen in psychiatry (Suh & Gallo, 1997; Vuorilehto et al., 2007). The prevalence of depression is lower in general practice than in psychiatry and screening identifies more false positives there (Mitchell & Coyne, 2010). This could also mean that there is lower agreement with rating scales for assessing severity in general practice than in psychiatry.

Corresponding to the findings of other authors, GPs felt uncomfortable with the threshold for diagnosing depression and considered a cut off level on a diagnostic scale to be arbitrary (Kendrick, 2000; Schumann et al., 2012). Embracing context was seen as important (Gask et al., 2008). In line with Armstrong and Earnshaw, we found that GPs tended to view depression as being caused by “problems of living” and therefore often identified as a “subtext” in the consultation (Armstrong & Earnshaw, 2004), or GPs moved away from the concept of depression as a disease, to focus more on the alleviation of suffering (Dowrick et al., 2009). This is in contrast to the way depression was handled by psychiatrists, who seemed to view both context and social conditions as the “subtext.”

The GPs often found it difficult to label a condition which was mixed with social problems. This corresponds with the findings of Chew-Graham et al. (2002) who reported that GPs perceive depression as a reaction to life events or change, and that they consider conventional clinical interventions of limited effectiveness in sociodeprived areas. Social deprivation or entrapment in aversive situations has been shown to be involved in chronic conditions in general practice (Brown et al., 2010; Kendrick, 2000; Macdonald et al., 2009).

Railton, Mowat, and Bain (2000) found that GPs and specialists respond in different ways to depression and inhabit different worlds in relation to the nature of their roles. The psychiatric world is focused on the specific diagnosis and treatment of illness, whereas general practice is more focused on the context and wider history of the presented problem. GPs focus on the individual patients and their stories. They look at the bigger picture, of which the patient is a part, and describe the narrative complexity of the patient’s problems (Davidsen & Reventlow, 2011). In the present study, GPs and psychiatrists talked in completely different narrative styles indicating their different worlds and understandings.

## Methodological considerations

In this qualitative study, knowledge was generated in an interaction between method, researcher, and informants (Järvinen & Mik-Meyer, 2005; Spradley, 1979). We strived at reflexivity throughout the entire research process, which means that we constantly took up an attitude of attending systematically to the context of knowledge construction, especially to the effect of the researcher, at every step of the research process (Malterud, 2001, 2002). In the phenomenologically inspired analysis, we tried, throughout the process, to be aware of and bracket our own preconceptions.

The first author carried out the interviews and the primary analysis. She is herself a physician with clinical experience both from general practice and psychiatry. This, we think, has led to having no preference for one of the groups and has diminished the tendency to identify more with one group than the other. She took several steps to ensure the validity during the interviews. She took a neutral, curious position and showed no bias toward any of the groups. She felt well received by the informants, felt a good rapport during the interviews, an honest atmosphere, and a willingness to become immersed in the subject. The participants were very open-minded and reflective about their own approach during the interviews. Although the same interview guide and the same interviewing style were used, the two groups of participants gave different types of descriptions, used different language, and had different reflections on their own way of thinking and working.

The analysis was carried out using IPA which states that it is in debt to symbolic interactionism (Smith, 1996) but still describes its view on interactionism as a concern about how meanings are constructed by individuals within both a social and personal world, which gives the term “interaction” the significance that meaning is a relational phenomenon that is constituted situationally incorporating the context (Järvinen & Mik-Meyer, 2005). The interview situation can be seen as having created two different types of interactions in the two settings but with the same engagement into the topic from both groups. We view the IPA, as it is described recently (Smith & Osborn, 2003; Smith et al., 2009), as also representing a complexity sensitive way of thinking. The advantage of IPA is the manageable procedure, although Smith stresses (Smith & Osborn, 2003) that there is no recipe. The different steps of the method were, however, applied systematically.

## Implications

In the proposed CC model, the psychiatric diagnosis of depression is supposed to form the basis of treatment. The diagnostic process may be difficult in primary care where patients present primarily with somatic symptoms (Goldberg, 1995; Kroenke, 2003; Simms, Prisciandaro, Krueger, & Goldberg, 2012; Tylee & Gandhi, 2005; Williamson & Yates, 1989) and where the symptoms are embedded in a complex life-historical and individual context, in which the GP is already involved (Van Weel-Baumgarten, Van den Bosch, Van den Hoogen, & Zitman, 2000). GPs are generalists and they cannot limit their focus to a well-defined number of patients’ symptoms but must take care of the range of problems and symptoms the patients present (Stange, 2009).

Effective implementation of a CC model depends on coherence and common understanding of depression and of how it should be dealt with (May, 2013). GPs and psychiatrists seem to have different views on what depression is, the work that has to be done to treat it, and the incorporation of this work into social and organizational practices (May et al., 2009). In addition, GPs feel that psychiatrists have the right of definition, which could possibly lead to a clash of interests. These different ways of understanding depression could possibly form a challenge to the cooperation between sectors. A UK study of a CC model showed less effect than previous US studies (Richards et al., 2013) and led to increased fragmentation of patient treatment, in opposition to the idea of collaborative care (Knowles et al., 2013). The authors point to that there is a need to focus more on organizational and managerial aspects to find a suitable and effective model. Our findings provide fertile ground for such organizational research into the actual implementation of cooperation between sectors to explore how differences are dealt with. In the context of the Danish CC study, we do not know if psychiatric nurses have the same understanding of depression as psychiatrists, nor how nurses will manage to navigate in this field. A study of nurses’ understanding as well as the implementation research could have relevance, not just for the treatment of depression, but also for CC models in other health care areas. In addition, the results set the stage for empirical research into the condition of depression in general practice and in psychiatry.

## Note

1 Present State Examination is a semi-structured diagnostic Interview tied-up with ICD-10.
